# Phycobiliproteins from *Arthrospira Platensis* (Spirulina): A New Source of Peptides with Dipeptidyl Peptidase-IV Inhibitory Activity

**DOI:** 10.3390/nu12030794

**Published:** 2020-03-18

**Authors:** Yuchen Li, Gilda Aiello, Carlotta Bollati, Martina Bartolomei, Anna Arnoldi, Carmen Lammi

**Affiliations:** Department of Pharmaceutical Sciences, University of Milan, 20133 Milan, Italy; yuchen.li@unimi.it (Y.L.); gilda.aiello@unimi.it (G.A.); carlotta.bollati@unimi.it (C.B.); martina.bartolomei@studenti.unimi.it (M.B.); anna.arnoldi@unimi.it (A.A.)

**Keywords:** allophycocyanin, bioactive peptides, C-Phycocyanin, DPP-IV, LC-MS/MS, *Spirulina platensis*

## Abstract

*Arthrospira platensis* (spirulina) is a cyanobacterium, which contains mainly two phycobiliproteins (PBP), i.e., C-phycocyanin (C-PC) and allophycocyanin (APC). In this study, PBP were hydrolyzed using trypsin, and the composition of the hydrolysate was characterized by HPLC-ESI-MS/MS. Furthermore, the potential anti-diabetic activity was assessed by using either biochemical or cellular techniques. Findings suggest that PBP peptides inhibit DPP-IV activity in vitro with a dose-response trend and an IC_50_ value falling in the range between 0.5 and 1.0 mg/mL. A lower inhibition of the DPP-IV activity expressed by Caco-2 cells was observed, which was explained by a secondary metabolic degradation exerted by the same cells.

## 1. Introduction

*Arthrospira platensis* (spirulina) is a cyanobacterium, which contains mainly two phycobiliproteins (PBP), namely C-phycocyanin (C-PC) and allophycocyanin (APC) [1]. Both phycobiliproteins are water-soluble, brightly colored, and highly fluorescent, and are used for numerous applications, such as fluorescent markers in biomedical research [2], nutrient ingredients, and natural dyes for food and cosmetics [3], as well as potentially therapeutic agents in oxidative stress-induced diseases [4]. Recently, some angiotensin converting enzyme (ACE) inhibitory peptides have been described, derived from either C-PC or APC [5,6]. Moreover, numerous biological effects of C-PC have been reported, such as anti-inflammatory [7], anti-apoptotic [8], antioxidant [9], and hypolipidemic [10] activities. In addition, the literature provides some evidence on the in vivo hypoglycemic activity exerted by C-PC peptides in different animal models [11,12]. In particular, a recent study has shown that C-PC peptides activate the insulin signaling pathway and glucokinase expression in the pancreas and liver of diabetic mice [13]. Moreover, the treatment of insulin-secreting INS-1 β-cells, a common model for diabetes research, with C-PC peptides induces an interesting protective mechanism [14]. Briefly, C-PC was capable to protect INS-1 pancreatic β-cells against methylglyoxal-induced cell dysfunction through modulating the PI3K/Akt pathway and the downstream FoxO1, which leads to the improvement of insulin secretion. 

Dipeptidyl peptidase IV (DPP-IV) (EC 3.4.14.5), a serine exopeptidase belonging to the prolyl oligopeptidase family, is considered an interesting therapeutic target for the management of type 2 diabetes (T2D), because it plays a key role in glucose metabolism by the N-terminal truncation and inactivation of the incretins glucagon-like peptide 1 (GLP-1) and gastrointestinal insulinotropic peptide (GIP) that together are responsible for up to 70% of post-prandial insulin secretion [15]. The inhibition of DPP-IV represents a new strategy for T2D treatment, and some therapeutic agents, also known as glyptins, are already available on the market. However, there is also lively research activity on food peptides as innovative DPP-IV inhibitors, since several studies have demonstrated that the ingestion of food protein-derived hydrolysates and peptides inhibits DPP-IV activity, either in vitro or in small animal models [16,17]. However, no specific DPP-IV inhibitory hydrolysates have been submitted to clinical studies for investigating their anti-diabetic properties, suggesting that human interventions should be performed in the near future in order to demonstrate the relevance of current research. 

Considering that there is an increasing interest in underexploited sustainable sources of proteins, such as microalgae, in a preceding paper, we investigated the inhibitory activity of a peptic and a tryptic hydrolysate from a total protein extract from spirulina and observed that, in vitro, the tryptic hydrolysate is a much better inhibitor of DPP-IV activity (IC_50_ = 0.1 mg/mL) than the peptic hydrolysate (IC_50_ = 3.4 mg/mL) [18]. In view of these results, the present study was aimed at investigating the potential ability of a tryptic PBP hydrolysate to modulate DPP-IV activity. The first objective was the optimization of PBP hydrolysis using trypsin and the characterization of the hydrolysate composition by HPLC-ESI-MS/MS analysis. The second objective was the evaluation of the potential anti-diabetic activity, which was carried out by measuring the effects on in vitro DPP-IV activity, as well as by performing experiments on Caco-2 cells, in order to measure the reduction of the enzymatic activity expressed on the cellular membranes of human enterocytes [19].

## 2. Materials and Methods

### 2.1. Reagents

All chemicals and reagents were of analytical grade. LC-grade H_2_O (18 MΩ cm) was prepared with a Milli-Q H_2_O purification system (Millipore, Bedford, MA, USA). Acetonitrile (ACN), ammonium bicarbonate, and trypsin from bovine pancreas (T1426, lyophilized powder, ≥ 10,000 BAEE unit/mg protein) were provided by Sigma-Aldrich (St. Louis, MO, USA). Bovine serum albumin (BSA) and β-mercaptoethanol were from Thermo Fisher Scientific (Life Technology, Milan, Italy). The Mini-Protean apparatus, Precision Plus protein standards, Bradford reagent, and Coomassie Blue G-250 were purchased from Bio-Rad (Hercules, CA, USA). 

### 2.2. Microalgae Biomass

Purified PBP dry powder was provided by Fuqing King Dnarmsa Spirulina CO., LTD. (Fujian, China), and was a material that had been purified and concentrated by microfiltration and ultrafiltration from crude protein extracts from *A. platensis*, cultivated in photoautotrophic conditions in an outdoor runway pool. Spray drying technology had been applied by the manufacturer to produce both the dry spirulina powder and dry PBP powder. The purity of the PBP powder was analyzed in advance to support the validity of the study, as reported in Figure S1.

### 2.3. Enzymatic Hydrolysis of PBP

The protease used for the hydrolysis was trypsin from bovine pancreas. Pure PBP (0.4 g) was suspended in 40 mL of Milli-Q H_2_O, the pH was adjusted to 8 with 1 M NaOH, and trypsin was added in a 1:50 ratio of E/S (*w*/*w*). The reaction mixture was then mixed and incubated at 37 °C for different times. The sample (40 μL) was pipetted out for the immediate blocking of the reaction at the 0, 5, 10, 20, 40, 60, 120, 180, and 210 min incubation time points. In this non-physiological way, the in vitro tryptic digestion of PBP was kept going overnight (16 h). After that, all of the hydrolyses were blocked by heating at 95 °C for 5 min, and then they were passed through ultrafiltration (UF) membranes (with a molecular weight (MW) cut-off of 3 kDa) using a Millipore UF system (Millipore, Bedford, MA, USA). All of the recovered peptides were lyophilized and stored at −80 °C until use. The degree of hydrolysis (DH) was measured by the o-phthaldialdehyde (OPA) assay, following a procedure previously described [20].

The SDS-PAGE analyses were performed on gels composed of a 4% polyacrylamide stacking gel (125 mM Tris-HCl, pH 6.8, 0.1%, m/v, SDS) over a 12% resolving polyacrylamide gel (375 mM Tris-HCl, pH 8.8, 0.1%, m/v, SDS buffer). Electrophoresis was performed with a Mini-Protean III vertical apparatus (Bio-Rad Lab, Hercules, CA, USA) at 100 V until the dye front reached the gel bottom. The resolved protein bands were stained by immersing the gel in a solution containing 45% methanol, 10% glacial acetic acid, and 0.25% Coomassie Brilliant Blue R-250. To visualize the bands, the gels were destained in a solution containing 45% methanol and 10% glacial acetic acid until they were clearly visible.

### 2.4. Analysis of the Hydrolysate by LC-ESI-MS/MS

The lyophilized tryptic hydrolysate was reconstituted in 500 μL of water solution containing 2% ACN and 0.1% formic acid, and 4 μL were injected into a HPLC-Chip system equipped with a SL IT mass spectrometer (Agilent Technologies Inc., Palo Alto, CA, USA). Each sample was loaded onto a 40 nL enrichment column (Zorbax 300SB-C18, 5 μm pore size), and separated onto a 43 mm × 75 μm analytical column (Zorbax 300SB- C18, 5 μm pore size). The separations were carried out in gradient mode at a flow rate of 500 nL/min. The elution solvent A was 95% water, 5% ACN, and 0.1% formic acid; solvent B was 5% water, 95% ACN, and 0.1% formic acid. The nano pump gradient program was as follows: 5% solvent B (0 min), 50% solvent B (0–50 min), 95% solvent B (50–60 min), and back to 5% for 10 min.

Data acquisition occurred in positive ionization mode. The capillary voltage was −2000 V, with an endplate offset of −500 V. Full scan mass spectra were acquired in the mass range from m/z 300 to 2000 Da. LC-MS/MS analysis was performed in data-dependent acquisition AutoMS(n) mode. In order to increase the number of identified peptides, three technical replicates (LC-MS/MS runs) were run for each hydrolysate. The MS/MS data were analyzed by the Spectrum Mill Proteomics Workbench (Rev B.04.00, Agilent), consulting the *A. platensis* database (12,530 entries) downloaded from UniProtKB/Swiss-Prot - ExPASy. Trypsin was selected as the cutting enzyme, two missed cleavages were allowed for each enzyme used, and the peptide mass tolerance was set to 1.0 Da and the fragment mass tolerance to 0.8 Da. An auto-validation strategy for both the peptide and protein polishing modes was performed using a false discovery rate (FDR) cut-off ≤1.2%.

### 2.5. In Vitro DPP-IV Activity of Tryptic PBP Peptides

The in vitro experiments were carried out in triplicate in a half volume 96 well solid plate (white). Each reaction (100 μL) was prepared by adding the reagents in a micro centrifuge tube in the following order: 1 × assay buffer [20 mM Tris-HCl, pH 8.0, containing 100 mM NaCl, and 1 mM EDTA]; PBP hydrolysate with a final concentration of 0.1, 0.5, 1.0, 2.5, or 5.0 mg/mL, or vehicle; and finally, the purified human DPP-IV enzyme (10 μL, from Cayman Chemical Company, Ann Arbor, MI, USA). Subsequently, the samples were mixed, and 50 μL of each reaction were transferred into each plate well. Each reaction was started by adding 50 μL of substrate solution (200 µM H-Gly-Pro-7-amido-4-methylcoumarin (AMC)) to each well and incubated at 37 °C for 30 min. Fluorescence signals were measured using the Synergy H1 fluorescent plate reader (Biotek, Bad Friedrichshall, Germany) (excitation and emission wavelengths 360 and 465 nm, respectively).

### 2.6. Cell Culture

Caco-2 cells, obtained from INSERM (Paris, France), were routinely sub-cultured at 50% density [21], and were maintained at 37 °C in a 90% air, 10% CO_2_ atmosphere, in Dulbecco Minimum Essential Medium (DMEM) containing 25 mM glucose, 3.7 g/L NaHCO_3_, 4 mM stable L-glutamine, 1% nonessential amino acids, 100 U/L penicillin, and 100 µg/L streptomycin (complete medium), supplemented with 10% heat-inactivated fetal bovine serum (FBS Hyclone Laboratories, Logan, UT, USA).

### 2.7. MTT Assay

A total of 3 × 10^4^ Caco-2 cells/well were seeded in 96-well plates and treated with 0.1, 0.5, 1.0, 2.5, and 5 mg mL^−1^ of tryptic PBP peptides and/or vehicle (H_2_O) in complete growth medium for 48 h at 37 °C under a 5% CO_2_ atmosphere, following the procedure previously reported [22]

### 2.8. Evaluation of the Inhibitory Effect of PBP Peptides on the In Situ DPP-IV Activity Expressed by Caco-2 Cells

A total of 5 × 10^4^ Caco-2 cells/well were seeded in black 96-well plates with clear bottoms. The second day after seeding, spent medium was discarded, and Caco-2 cells were treated with 0.1, 0.5, 1.0, 2.5, and 5 mg/mL of tryptic PBP peptides and/or vehicle in growth medium for 30 min and 1, 3, 6, and 24 h at 37 °C. Afterwards, the treatments were removed, and the Caco-2 cells were washed once with 100 µL of PBS w/o Ca^++^ and Mg^++^. Thus, 40 µL of Gly-Pro-AMC substrate at the concentration of 20.0 µM in PBS w/o Ca^++^ and Mg^++^ were added into each well, and the fluorescence signals (ex./em. 350/450 nm) were measured using the Synergy H1 microplate reader every 1 min for 10 min.

### 2.9. Statistical Analysis

All measurements were performed in triplicate and the results are expressed as the mean ± standard deviation (SD) of six independent experiments, each performed in triplicate, where p-values < 0.05 were considered to be significant. Statistical analyses were performed by one-way and two-way ANOVA (Graphpad Prism 8.3, GraphPad Software, La Jolla, CA, USA), followed by Dunnett’s and Tukey’s tests, respectively.

## 3. Results

### 3.1. PBP In Vitro Digestion and Peptide Identification by Nano ESI-MS/MS

**Figure 1:**
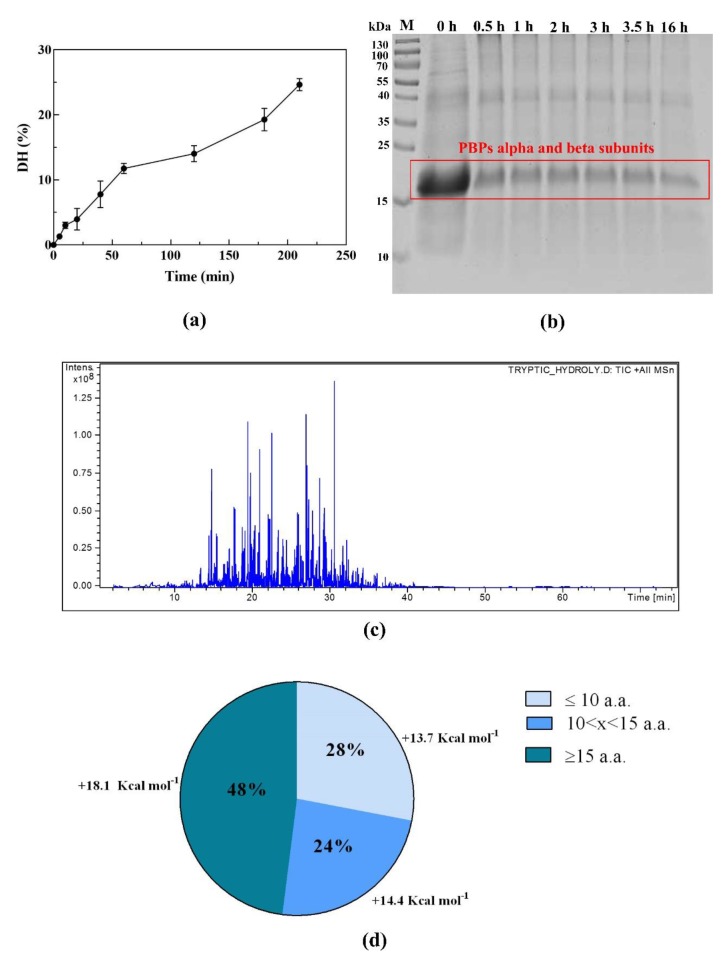
The preparation and analysis of the tryptic PBP hydrolysate. (**a**) The trend of degree of hydrolysis (DH) versus time in the first 3.5 h of digestion with trypsin. (**b**) The SDS-PAGE analysis of the hydrolysis sampled at different time points. (**c**) The total ion chromatogram (TIC) of the phycobiliprotein (PBP) hydrolysate. (**d**) The length and hydrophobicity distribution of the peptides from the PBP hydrolysate.

In order to release the peptides with potential health benefits from PBP, a non-physiological in vitro digestion was performed using trypsin. Figure 1a shows the increasing trend of DH in the first 3.5 h of reaction, which reached a value of 24.7%, suggesting an efficient digestion. The process was continued overnight (16 h), reaching a final DH value of 47.0% (data not shown). The SDS-PAGE analysis (Figure 1b) indicated that the PBP band—including the C-PC alpha and beta subunits, as well as the APC alpha and beta subunits, all of which have MWs of 17–18 kDa—was less intense at the end than at the beginning of hydrolysis (time 0 h), even though this band was still detectable after 16 h of treatment.

The peptide composition of PBP hydrolysates was analyzed by LC-ESI-MS/MS. Figure 1c presents the total ion chromatogram (TIC) of the sample, whereas the overall identified peptides are reported in Table S1. In total, 26 unique sequences were identified as peptides produced by the tryptic hydrolysis. The length of those peptides ranged from 7 to 27 amino acids. Among these, seven were from the C-PC alpha chain, six from the C-PC beta chain, and five and eight from the APC alpha and beta subunits, respectively. The sequence coverages (%AA) of each identified protein were 65.4%, 45.3%, 41.6%, and 65.8%, respectively. Based on the MS/MS results, the length distribution of peptides derived from the tryptic PBP hydrolysate (Figure 1d) and the average hydrophobicity of peptides in each subgroup were calculated (Figure 1d). The peptides composed of 7–10 amino acids accounted for 28% of the PBP hydrolysate and had an average hydrophobicity of 13.7 kcal mol^−1^, the peptides containing 11–14 amino acid residues accounted for 24% of the hydrolysate and had an average hydrophobicity of 14.4 kcal mol^−1^, and the peptides with a length of 15–27 amino acids were 48% of the total and had an average hydrophobicity of 18.1 kcal mol^−1^.

### 3.2. PBP Peptides Inhibit DPP-IV Activity In Vitro and at the Cellular Level

**Figure 2:**
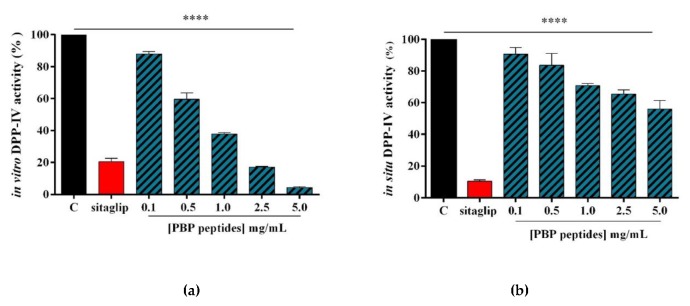
Evaluation of the inhibitory activity of the tryptic PBP hydrolysate on DPP-IV: (**a**) the in vitro inhibition of the activity of human recombinant DPP-IV; (**b**) the in situ inhibition of the DPP-IV activity expressed by non-differentiated Caco-2 cells, after 30 min of treatment. The data are represented as the means ± SD of six independent experiments, performed in triplicate. Statistical analysis was performed by one-way ANOVA, followed by Dunnett’s test (****) *p* < 0.0001.

In order to assess the ability of tryptic PBP peptides to modulate DPP-IV activity, preliminary in vitro experiments were performed using the purified recombinant DPP-IV enzyme. The enzyme was incubated with the PBP peptides in the concentration range of 0.1–5.0 mg/mL and the fluorescent substrate, H-Gly-Pro-AMC, for 30 min at 37 °C. The reaction was monitored by measuring the fluorescence signals (465 nm) due to the release of the free AMC group after the cleavage of the peptide H-Gly-Pro, catalyzed by DPP-IV. Sitagliptin was used as a reference compound. Figure 2a shows that the tryptic PBP hydrolysate reduces DPP-IV activity in vitro by 11.9% ± 2.8%, 40.5% ± 7.6%, 62.1% ± 1.3%, 82.9% ± 0.7%, and 95.8% ± 0.3%, at 0.1, 0.5, 1.0, 2.5, and 5.0 mg/mL, respectively, whereas sitagliptin (1 µM) inhibits the enzyme activity by 79.5% ± 2.5%.

The inhibitory activity was then evaluated in situ using Caco-2 cells, which express high levels of DPP-IV on their cellular membranes [19]. Firstly, in order to exclude any potential cytotoxic effects exerted by PBP peptides, MTT experiments were performed after 48 h of cellular treatment in a 0.1–5.0 mg/mL concentration range. The results of this experiment showed that the PBP hydrolysate was safe for the Caco-2 cells at each dose tested (data not shown). Therefore, the cell experiments for measuring the in situ DPP-IV activity were carried out at the same concentrations applied in the biochemical assay. Namely, Caco-2 cells were treated with PBP peptides in the range of concentrations 0.1–5.0 mg/mL, and their effects on cellular enzyme activity were evaluated using the same fluorescent substrate for 30 min at 37 °C, and again, sitagliptin was used as a reference compound. Figure 2b indicates that the PBP hydrolysate inhibited cellular DPP-IV activity by 9.3% ± 4.1%, 16.4% ± 7.4%, 29.2% ± 1.3%, 34.6% ± 2.7%, and 44% ± 5.4%, at 0.1, 0.5, 1, 2.5, and 5 mg/mL, respectively, whereas sitagliptin did such by 89.6% ± 0.9% at 1.0 µM.

### 3.3. Kinetics of the Inhibition of DPP-IV Activity Expressed by Caco-2 Cells Induced by the PBP Hydrolysate

**Figure 3:**
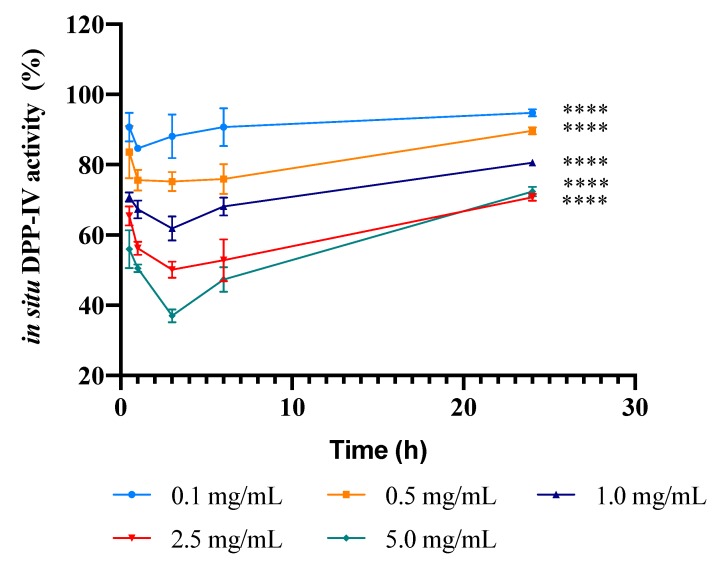
The kinetics of the inhibition of in situ DPP-IV activity after incubating Caco-2 cells with the PBP hydrolysate at different concentrations. The data are represented as the means ± SD of six independent experiments, performed in triplicate. Multiple comparisons by two-way ANOVA were performed, comparing within each concentration, each time point, and comparing within each time point, each concentration. (****) *p* < 0.0001.

In order to evaluate the kinetics of DPP-IV inhibition by PBP peptides in a cellular system, Caco-2 cells were treated with the PBP hydrolysate at 0.1, 0.5, 1.0, 2.5, and 5.0 mg/mL for 0.5, 1, 3, 6, and 24 h. This experiment allowed us to evaluate the potential transient DPP-IV inhibitory nature of the PBP hydrolysate. At each time point, the sample inhibitory activity was assessed by measuring the fluorescence signals due to the release of the free AMC group after the cleavage of the peptide H-Gly-Pro, catalyzed by cellular DPP-IV. Figure 3 shows the curves of the dependence of the enzyme activity on time, obtained at the different concentrations of the PBP hydrolysate. In detail, after 0.5 h incubation, the hydrolysate inhibited DPP-IV activity by 9.3% ± 4.1%, 16.4% ± 7.4%, 29.2% ± 1.3%, 34.6% ± 2.7%, and 44% ± 5.4%; after 1 h of incubation by 15.3% ± 0.3%, 24.4% ± 2.9%, 32.7% ± 2.5%, 43.7% ± 1.9%, and 49.5% ± 1.1%; after 3 h by 11.9% ± 5.7%, 24.8% ± 2.5%, 38.1% ± 3.1%, 49.9% ± 2.0%, and 63.1% ± 1.6%; after 6 h by 9.3% ± 5.4%, 24.1% ± 4.3%, 32% ± 2.6%, 47.2% ± 6.0%, and 52.7% ± 3.5%; and after 24 h by 6.3% ± 0.3%, 10.4% ± 0.1%, 18.7% ± 0.02%, 28.3% ± 0.6%, and 27.9% ± 0.5%; at 0.1, 0.5, 1.0, 2.5, and 5.0 mg/mL, respectively. The maximum reductions of DPP-IV activity were observed at around 1 h for the treatments at 0.1 and 0.5 mg/mL, and at around 3 h for the treatments at 1.0, 2.5, and 5.0 mg/mL.

## 4. Discussion

For the first time, this study provides new evidence regarding the ability of tryptic PBP to modulate DPP-IV activity in vitro on human recombinant enzyme and in situ on the cellular intestinal membrane level. In a previous study, we reported the DPP-IV inhibitory activity of a tryptic hydrolysate derived from a spirulina total protein extract [18]. In total, 78 peptides were identified, out of which 19 individual sequences were attributed to C-PC or APC, i.e., four to the C-PC alpha subunit, four to the C-PC beta subunit, five to the APC alpha subunit, and six to the APC beta subunit, corresponding to sequence coverages of 37.6%, 35.4%, 41.6%, and 49.6%, respectively. The sequence coverages of identified C-PC alpha and beta subunits, and APC alpha and beta subunits from PBP digests were higher than those obtained from spirulina total protein hydrolysates, obviously reflecting that the accuracy of the LC-MS/MS analysis was improved after PBP purification. In the same study, it was also shown that the spirulina hydrolysate exerts a potential hypoglycemic effect, targeting DPP-IV activity either in vitro or in a cellular system [18]. Those results stimulated the present investigation focused on a tryptic PBP hydrolysate. The chemical analysis of this material permitted the detection of 26 peptides, out of which 13 peptides were assigned to C-PC and 13 to APC (Table S1), reflecting the specificity of trypsin, which generally cleaves peptide chains at the carboxyl side of lysine or arginine. Interestingly, all of the peptides previously identified in the spirulina hydrolysate were also detected in the PBP hydrolysate. In addition, the evaluation of the PBP peptide distribution as a function of length and hydrophobicity may be doubly useful. Firstly, hydrophobicity may positively contribute to peptide bioavailability after oral ingestion. Even though the molecular mechanism involved in food bioactive peptide absorption across the intestinal epithelium has not yet been elucidated, transcytosis is an energy-dependent transcellular transport pathway, which favors the transport of bioactive peptides with long chains and high hydrophobicity [23]. Moreover, it has also been established that hydrophobic peptides are more prone to exert inhibitory activity against DPP-IV [17].

Discussing now the inhibitory effects on DPP-IV activity exerted by the tryptic PBP hydrolysate, the in vitro screening suggested that it inhibits the enzyme with a dose-response trend, and an IC_50_ value between 0.5 and 1.0 mg/mL (Figure 2a). This means that the tryptic PBP hydrolysate is about three-fold more active than the tryptic spirulina hydrolysate, which reduces the enzyme activity by 55.3% at 2.5 mg/mL. Since 19 peptides out of the 26 peptides identified in the PBP hydrolysate had been also identified in the spirulina hydrolysate, it seems reasonable to formulate the hypothesis that the PBP peptides have a relevant role in the DPP-IV inhibitory activity of the spirulina hydrolysate, whose lower activity may probably be explained by the unfavorable effects of numerous inactive or poorly active peptides present in its complex composition.

This PBP hydrolysate is more active than the previously reported tryptic hydrolysates, such as those from soybean and hempseed. In fact, the soybean hydrolysate inhibits DPP-IV activity by 15.3% ± 11.0% and 11.0% ± 0.30% at 1.0 and 2.5 mg/mL, respectively [24], whereas the hempseed does so by 32.0% ± 6.2% at 1.0 mg/mL [25]. Our results are also in line with the activities of other food protein hydrolysates [26], such as of marine, plant, bovine meat, and egg proteins, which have been widely investigated as sources of DPP-IV inhibitory peptides. Particularly good results have been obtained especially with milk proteins, since a caprine casein hydrolysate, a bovine milk protein isolate, and a camel milk hydrolysate have shown IC_50_ values equal to 0.8, 0.8, and 0.5 mg/mL, respectively [27,28,29].

However, it is important to underline that all of these studies were carried out exclusively using biochemical tools based on purified enzyme. This traditional approach doubtlessly represents a great limitation for a more realistic characterization of the hydrolysates with DPP-IV inhibitory activity. In light of these observations, a specific feature of our work was the employment of an intestinal cell-based assay for measuring the enzymatic activity, which represents a complementary and cost-effective strategy for a more efficient discovery of food-derived DPP-IV inhibitors [30]. In fact, the enterocyte luminal surface expresses a great quantity of DPP-IV: this means that, before absorption, any potential inhibitor deriving from food digestion is likely to interact with intestinal DPP-IV and other intestinal peptidases. This exposes the peptides to the risk of secondary metabolic degradation, which may dynamically modify the composition of the hydrolysate, modulating its intrinsic bioactivity. In the case of an extensive secondary metabolic degradation, a drastic reduction of the bioactivity might also take place. In order to check this hypothesis, Caco-2 cells were treated with the same PBP hydrolysate concentrations used in the in vitro test (0.1–5 mg/mL) for 30 min at 37 °C. The results indicate that even though a certain bioactivity is maintained, a significant reduction is observed (Figure 2b). This outcome may be explained by considering that the metabolic effects exerted by Caco-2 cells cleave the PBP peptides into shorter sequences that are characterized by a reduced activity. A similar metabolic degradation and consequent reduction of DPP-IV inhibition have been observed when testing the spirulina hydrolysate at the cellular level [18].

This hypothesis is reinforced by the kinetic study of the in situ DPP-IV activity after incubation of the Caco-2 cells with the PBP hydrolysate at different concentrations as a function of time (Figure 3). As expected, the PBP hydrolysate reduces the cellular DPP-IV activity with an effectiveness that depends on the concentration. Interestingly, the curves at the lower doses are characterized by a maximum that is achieved after 1 h treatment, and those of the higher doses have a maximum that is achieved after 3 h of treatment (Figure 3). After the maximum, the secondary metabolic activity of Caco-2 cells prevails, and the changing composition of the hydrolysate has an unfavorable effect on the bioactivity. The susceptibility to gastrointestinal peptidase degradation is generally considered a weakness of bioactive food peptides. However, the maxima in these curves suggest that, at least at the beginning of the treatment, the secondary metabolic degradation exerted by intestinal cells may produce a favorable effect on the bioactivity, whereas only when the degradation is much more extensive does it induce a substantial limitation of the inhibitory activity.

In conclusion, this work provides new insights on the ability of tryptic PBP hydrolysate to modulate DPP-IV activity in Caco-2 cells. These results draw attention to the possibility of exploiting a tryptic PBP hydrolysate as a new source of peptides for the development of nutraceuticals or functional foods. Moreover, they underline the dynamic nature of bioactive food hydrolysates that may be modulated by the biological systems with whom they get in touch. In this direction, future investigations will be planned to simulate sequential human-like gastrointestinal digestion and the absorption of PBP and total spirulina peptides together, and further experiments will be carried out for confirming their biological activities as DPP-IV inhibitors, also assessing the effects on GLP-1 levels and stability.

## Supplementary Materials

The following are available online at https://www.mdpi.com/2072-6643/12/3/794/s1, Table S1: HPLC-Chip-ESI-MS/MS based identification of peptides in the tryptic PBP hydrolysate. Figure S1: SDS-PAGE of purified PBP and total spirulina protein extracts.

## Abbreviations

ACE	angiotensin converting enzyme
ACN	acetonitrile
AMC	7-Amido-4-methylcoumarin
APC	allophycocyanin
BSA	bovine serum albumin
C-PC	C-phycocyanin
DH	degree of hydrolysis
DMEM	Dulbecco Minimum Essential Medium
DMSO	dimethyl sulfoxide
DPP-IV	dipeptidyl peptidase IV
FBS	fetal bovine serum
GIP	insulinotropic peptide
GLP-1	glucagon-like peptide
HMGCoAR	3-hydroxy-3-methyl-glutaryl-coenzyme A reductase
HPLC-ESI-MS/MS	High-performance liquid chromatography electrospray ionization tandem mass spectrometry
INS-1 β-cells	rat insulinoma beta cells
MTT	3-(4,5-dimethylthiazol-2-yl)-2,5-diphenyltetrazolium bromide
MW	molecular weight
OPA	o-phthaldialdehyde
PBP	phycobiliproteins
PBS	phosphate buffered saline
SDS-PAGE	sodium dodecyl sulphate-polyacrylamide gel electrophoresis
T2D	type 2 diabetes
TIC	total ion chromatogram
UF	ultrafiltration
