# Supplementary material for: Phycobiliproteins from Arthrospira Platensis (Spirulina): A New Source of Peptides with Dipeptidyl Peptidase-IV Inhibitory Activity
Source: Nutrients. 2020 Mar 18;12(3):794. doi: 10.3390/nu12030794 (PMC7146380; doi:10.3390/nu12030794)
Supplement: Supplementary file 1 [file nutrients-12-00794-s001.pdf]

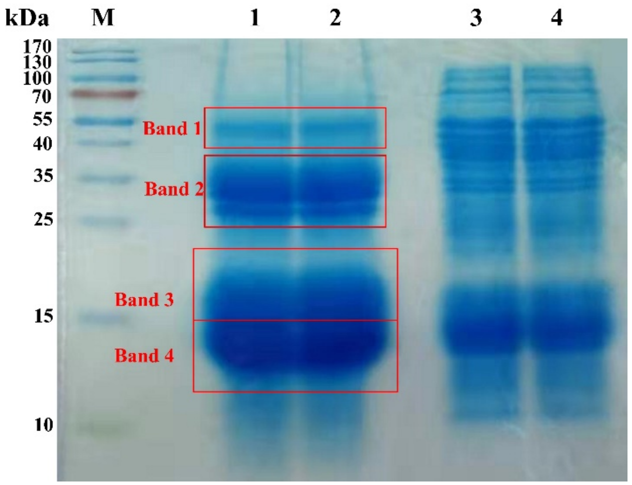

Figure S1. SDS-PAGE of purified PBP and total spirulina protein extracts. M-marker; Lane 1 and 2 - two parallels of purified PBP; 3 and 4 -two parallels of total spirulina protein extracts.

The PBP we used was obtained from Fuqing King Dnarmsa Spirulina CO., LTD., a company producing food-grade Spirulina powder and purifying PBP by techniques such as microfiltration and ultrafiltration on large scale. This PBP was not as pure as the analytical grade C-PC and APC but it satisfied our demand, since we aimed to understand the bioactivity of its peptides considering it as a dietary source. Before preparing the peptides, we evaluated the purity of PBP by SDS-PAGE and in-gel digestion. We loaded the same amount of protein samples to perform SDS-PAGE. As shown in Figure S1, compared with the lanes of total spirulina protein extracts, lanes of PBP presents less bands of unwanted protein but much more intense bands of PBP alpha and beta subunit (band 3 and 4, 17-18 kDa). After in-gel digestion and LC-MS/MS identification, we identified that band 3 and 4 were exactly the mixture of alpha and beta subunits from C-PC and APC. As to band 2, two subunits of C-PC and APC were also identified as the main proteins, from which we supposed band 2 was the PBP heterodimers composed of alpha and beta subunits (around 35 kDa). The present of heterodimers could be due to the uncompleted reduced reaction between the PBP and the reduced loading buffer, so that the thioether bonds linking alpha and beta subunits were not destroyed completely. A few unwanted proteins present in band 1 and 2 showed much lower spectrum intensity compared with that of PBP. Hence, we considered the PBP had a proper purity and met our requirement.

Table S1. HPLC-Chip-ESI-MS/MS based identification of peptides in the tryptic PBP hydrolysate.

No	Protein name [a]	Accession no [a]	m/z [b] (charge)	Start	MH^+ Matched (Da)	Peptide sequence [b]	Spectra (#)	Distinct Peptides (#)	Total Protein Spectral Intensity	% AA Coverage
1	C-phycocyanin alpha subunit	D5A5N9	846.28(3)	138	2536,20	(K)ANHGLSGDAAVEANSYLDYAINALS(-)	42	7	8.74e+09	65.4
			503.44(3)	48	1507,77	(K)ADSLISGAAQAVYNK(F)				
			994.71(2)	63	1987,89	(K)FPYTTQMQGPNYAADQR(G)				
			515.69(3)	3	1544,79	(K)TPLTEAVSIADSQGR(F)				
			727.97(2)	18	1454,76	(R)FLSSTEIQVAFGR(F)				
			842.49(2)	121	1683,86	(R)TFELSPSWYIEALK(Y)				
			899.45(1)	87	899,46	(R)DIGYYLR(M)				
2	C-phycocyanin beta chain	P72508	741.96(3)	16	2223,06	(R)GEMLSTAQIDALSQMVAESNK(R)	14	6	2.30e+09	45.3
			938.86(2)	92	1875,91	(R)YVTYAVFAGDASVLEDR(C)				
			703.25(2)	44	1404,74	(R)ITSNASTIVSNAAR(S)				
			602.8(3)	115	1805,97	(R)ETYLALGTPGSSVAVGVGK(M)				
			445.25(2)	85	889,48	(R)DMEIILR(Y)				
			569.45(3)	21	1705,84	(R)TAQIDALSQMVAESNK(R)				
3	Allophycocyanin alpha subunit	D5A426	904.04(3)	135	2709,26	(K)SVATSLLSGEDAAEAGAYFDYLIGAMS(-)	14	5	1.37e+09	41.6
			700.15(2)	118	1398,76	(K)SLGTPIEAVAEGVR(A)				
			523.42(2)	7	1045,53	(K)SIVNADAEAR(Y)				
			525.36(2)	17	1049,53	(R)YLSPGELDR(I)				
			479.24(2)	84	957,47	(R)DLDYYLR(L)				
4	Allophycocyanin beta chain	D5A425	598.62(3)	1	1792,88	(-)MQDAITSVINSSDVQGK(Y)	20	8	3.28e+09	65.8
			731.37(3)	114	2191,14	(K)ETYNSLGVPIGATVQAIQAMK(E)				
			514.74(2)	29	1027,52	(K)AYFATGELR(V)				
			594.36(2)	135	1187,63	(K)EVTAGLVGADAGK(E)				
			504.81(2)	18	1008,54	(K)YLDASAIQK(L)				
			479.24(2)	84	957,47	(R) DLDYYLR (Y)				
			629.41(3)	91	1885,90	(R)YATYAMLAGDPSILDER(V)				
			672.78(2)	40	1344,75	(R)AATTISANAANIVK(E)				
